# Actuating Shape Memory Polymer for Thermoresponsive Soft Robotic Gripper and Programmable Materials

**DOI:** 10.3390/molecules26030522

**Published:** 2021-01-20

**Authors:** Dennis Schönfeld, Dilip Chalissery, Franziska Wenz, Marius Specht, Chris Eberl, Thorsten Pretsch

**Affiliations:** 1 Fraunhofer Institute for Applied Polymer Research IAP, Geiselbergstr. 69, 14476 Potsdam, Germany; dennis.schoenfeld@iap.fraunhofer.de (D.S.); dilip.chalissery@iap.fraunhofer.de (D.C.); 2 Fraunhofer Institute for Mechanics of Materials IWM, Wöhlerstr. 11, 79108 Freiburg, Germany; franziska.wenz@iwm.fraunhofer.de (F.W.); MariusSpecht@web.de (M.S.); chris.eberl@iwm.fraunhofer.de (C.E.); 3 Department of Microsystems Engineering IMTEK, University of Freiburg, Georges-Koehler-Allee 078, 79110 Freiburg, Germany

**Keywords:** additive manufacturing, soft robotics, actuation, programmable materials, polyester urethane, shape morphing, unit cell

## Abstract

For soft robotics and programmable metamaterials, novel approaches are required enabling the design of highly integrated thermoresponsive actuating systems. In the concept presented here, the necessary functional component was obtained by polymer syntheses. First, poly(1,10-decylene adipate) diol (PDA) with a number average molecular weight *M*_n_ of 3290 g·mol^−1^ was synthesized from 1,10-decanediol and adipic acid. Afterward, the PDA was brought to reaction with 4,4′-diphenylmethane diisocyanate and 1,4-butanediol. The resulting polyester urethane (PEU) was processed to the filament, and samples were additively manufactured by fused-filament fabrication. After thermomechanical treatment, the PEU reliably actuated under stress-free conditions by expanding on cooling and shrinking on heating with a maximum thermoreversible strain of 16.1%. Actuation stabilized at 12.2%, as verified in a measurement comprising 100 heating-cooling cycles. By adding an actuator element to a gripper system, a hen’s egg could be picked up, safely transported and deposited. Finally, one actuator element each was built into two types of unit cells for programmable materials, thus enabling the design of temperature-dependent behavior. The approaches are expected to open up new opportunities, e.g., in the fields of soft robotics and shape morphing.

## 1. Introduction

Soft robotics, as well as the still novel metamaterials [1,2] or programmable materials [3], require compliant actuator materials. Shape memory polymers (SMPs) perfectly fulfill this criterion. SMPs are stimuli-responsive materials, which are able to fix a temporary shape after a thermomechanical treatment, also denoted as “programming”. The temporary shape will be stable until the one-way shape memory effect (1W SME) is triggered, whereupon the polymer almost completely returns into the permanent shape [4]. Shape recovery is an entropically driven process. It is based on the phenomenon of entropy elasticity; the theory of rubber elasticity provides the basis [5]. Most commonly, the shape memory effect is triggered by heat [4,6,7,8,9,10,11,12]. Alternatively, switching can be realized for materials equipped with appropriate fillers by indirect heating when applying an electric [13,14] or a magnetic field [15,16] or, e.g., by illumination with near-infrared light in the case of SMPs with photoresponsive fillers [17,18].

In the past few years, it has become known how to transfer semicrystalline SMPs into two metastable states, between which they can be switched back and forth virtually as often as desired by varying the temperature. The driving forces for the so-called two-way shape memory effect (2W SME) in polymers are phase transitions between crystalline and amorphous phases, supported by entropy elasticity as discovered by Mather’s group for cross-linked poly(cyclooctene) films in the presence of an external load [19]. Later, the programming technology was further developed and transferred to other polymer systems so that actuation in the stress-free state became possible [20,21,22,23]. As known from semicrystalline polyester urethane (PEU), actuation can also be very pronounced and complies with the same physical principles [24]. The fact that polymers react to temperature changes in their surroundings makes them interesting candidates for applications in which complex switching and control electronics are to be avoided [25]. For example, in the field of soft robotics, a specific subfield of robotics that deals with the construction of robots from highly compliant materials [26,27,28], first steps were taken to demonstrate the attractiveness of SMPs [29,30,31,32]. Anyway, there are only a few concepts in this respect, and the developments are primarily based on the 1W SME [33,34,35]. On the other side, an example for the 2W SME was provided by Behl et al. [22], who employed chemically cross-linked poly(ω-pentadecalactone) and poly(*ε*-caprolactone)-based polyester urethane to fabricate a gripper, which after programming opened at 50 °C and closed at 0 °C. The system was able to grab and release a small coin upon cooling and heating. In another work, Zhou et al. [36] introduced a gripper from a chemically cross-linked poly(octylene adipate) and demonstrated how programming enables the lifting and depositing of a small screw at temperatures between 10 °C and 36 °C. In both cases, the essential advantage of the two-way shape memory effect becomes clear: autonomous motion. However, both studies focus mainly on the handling of small and simple objects. They have in common that the selection of the lower actuation temperature necessitates active cooling since it is below room temperature. In addition, the complexity of actuation is not really significant.

To address these problems, this contribution reports on the synthesis of a PEU with promising thermal and mechanical properties. Furthermore, we describe a route of how to identify pronounced actuation and used thermomechanical treatment to facilitate reliable actuator functionality. On this basis, a novel system was built in which the actuator transfers its motion to the stiff, mobile components of a gripper, thus opening the door to grab and release bigger and more complex objects.

In order to pursue a new direction, a coupling of actuator elements with the elastic parts of a mechanical unit cell or with a unit cell, which was completely built from one elastomer, was realized. It will be demonstrated that such actuating unit cells can change their states as a function of temperature, thus symbiotically combining actuation with unit cell functionalities. The obtained shape morphing unit cells are considered as the first step toward the production of novel, thermoresponsive metamaterials, which are assembled out of periodically repeated unit cells that determine their physical properties [37]. In essence, it will be demonstrated that the ability of the polymer to transfer its movements makes it possible to produce completely new systems with programmable property profiles.

## 2. Results and Discussion

**Figure 1:**
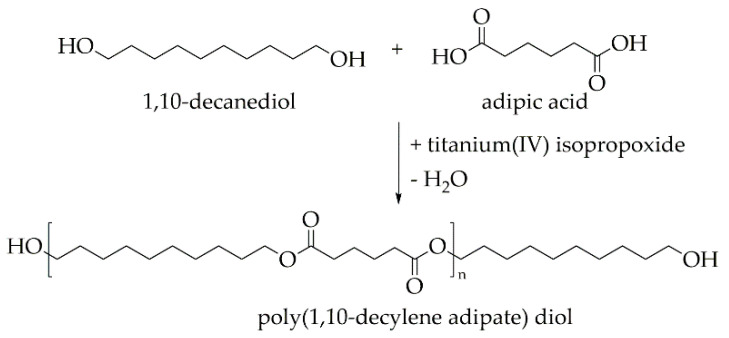
Synthesis of poly(1,10-decylene adipate) diol via polycondensation reaction.

Poly(1,10-decylene adipate) diol (PDA) is a promising building block both in the chemistry of polyester urethanes and polyester urethane ureas [25]. It was synthesized in the present work in a polycondensation reaction from 1,10-decanediol and adipic acid (Figure 1). 

**Figure 2:**
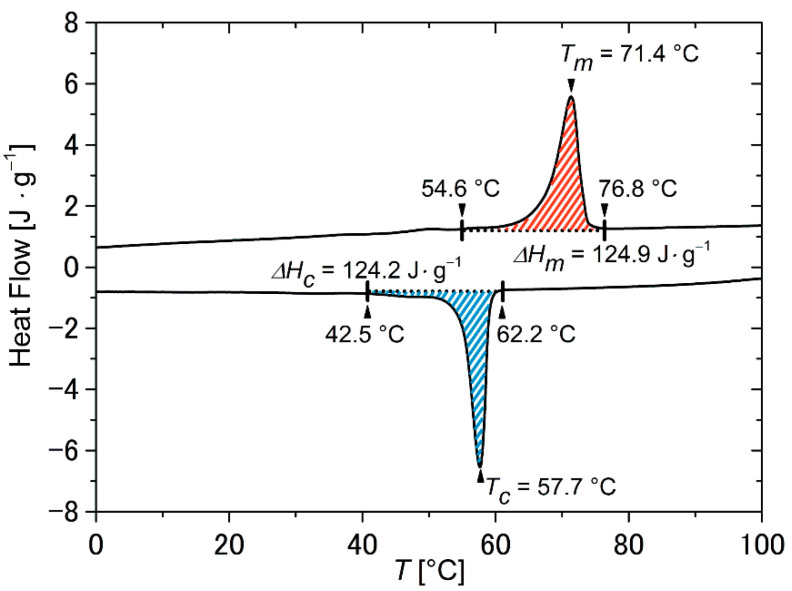
Differential scanning calorimetry (DSC) thermogram of poly(1,10-decylene adipate) diol showing the second heating and cooling with temperature rates of 10 °C·min^−^^1^. The enthalpies of melting *ΔH_m_* (red dashed area) and crystallization *ΔH_c_* (blue dashed area) are included.

The number average molecular weight *M*_n_ of the PDA was determined to ~3290 g·mol^−1^, and the calorimetric properties were characterized. The obtained polyester exhibits a melting transition spreading from 55 °C to 77 °C with a peak temperature of 71 °C and a crystallization transition ranging from 62 °C to 43 °C with a peak temperature located at 58 °C (Figure 2). Thus, both phase transition temperatures were well above room temperature. As expected, the assigned phase transitions were in good agreement with those of other aliphatic polyesters [38].

**Figure 3:**
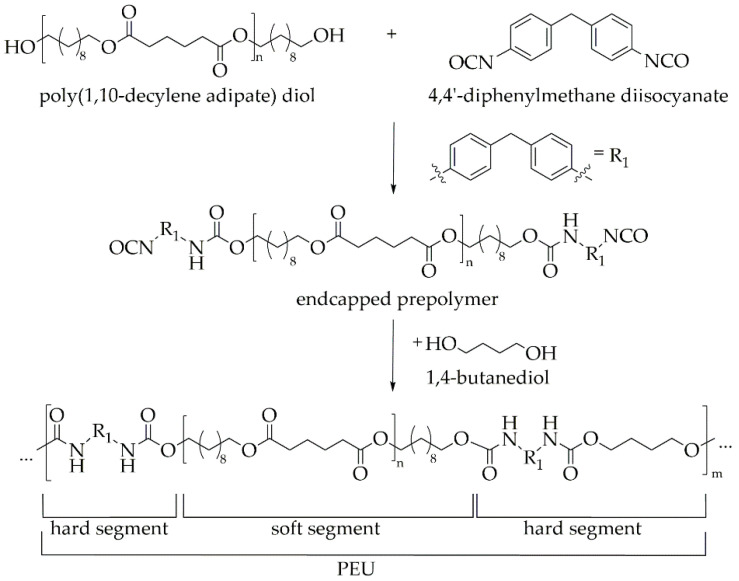
Synthesis of poly(1,10-decylene adipate) diol (PDA)-based polyester urethane (PEU) via polyaddition reaction using the prepolymer method.

Following the prepolymer method [39,40,41], the freshly synthesized PDA was brought to reaction with 4,4′-diphenylmethane diisocyanate (4,4′-MDI) in order to build up an isocyanate-endcapped prepolymer, before the chain extender 1,4-butanediol (BD) was finally added. This resulted in the formation of a polyester urethane (PEU, Figure 3). 

Herein, it is obvious that the so-called soft segment is composed of the synthesis building block PDA while the hard segment was obtained from the reaction of MDI and BD. Fourier-transform infrared (FT-IR) spectroscopy was used to verify the completeness of the polyaddition reaction. Since the characteristic vibrational modes for polyester urethane were visible and, in addition, only a very weak signal associated with freely available isocyanate appeared in the spectrum, the reaction was mostly complete. A detailed analysis can be found in the Supplementary Materials (Figure S1).

**Figure 4:**
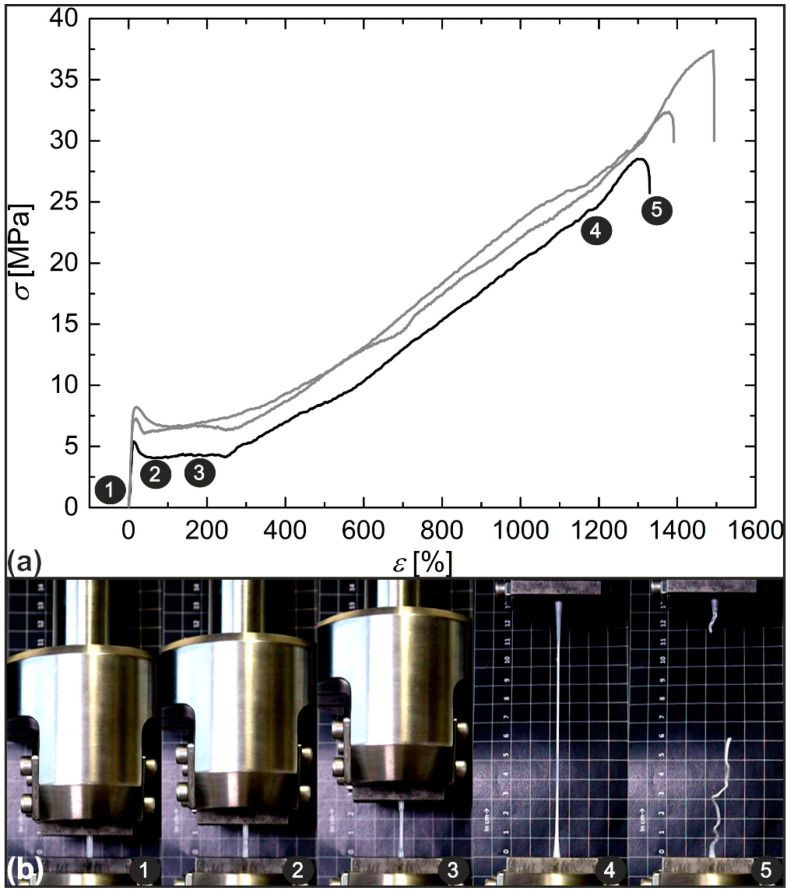
Mechanical characterization of PDA-based PEU using tensile tests: engineering stress–strain curves (**a**) and the associated deformation behavior (**b**). The experiments were carried out on tensile bars at 23 °C with an initial strain rate of 1%·min^−1^ until 5% of strain were reached and continued with 2000%·min^−1^ until rupture occurred.

Once characterized, the PEU was melt-extruded into a filament as essential for further processing via fused filament fabrication (FFF). For this purpose, the same extrusion line was used as reported recently [42]. The obtained filament had a homogenous diameter of 2.85 ± 0.08 mm, so that an important attribute for further processing was fulfilled. Tensile bars of type 5B according to ISO 527-2:1996 [43] were then additively manufactured, and their mechanical behavior was determined in tensile tests at ambient temperature (Figure 4).

From the stress–strain relationship, an averaged Young’s modulus of 258.7 ± 10.2 MPa was determined. In all three measurements, a yield-point occurred at a strain value of 17 ± 3%, corresponding to a stress of 7.1 ± 1.4 MPa (Figure 4a). Further increasing the load resulted in necking (Figure 4b, images 2–4) as already verified for similar materials [44,45,46] and strain softening first, followed by strain hardening as accompanied by an increase in stress, culminating in specimen rupture at strains of 1405 ± 83%. The material behavior can be explained with the coexistence of two types of PDA segments, at which one part was highly flexible and amorphous while the other one was rigid and crystalline. In the course of deformation, a progressive conversion from amorphous to crystalline segments seemed to occur. The assumption was supported by a whitish coloring of the tensile bar as associated with a crystallization process (Figure 4b, image 4).

**Figure 5:**
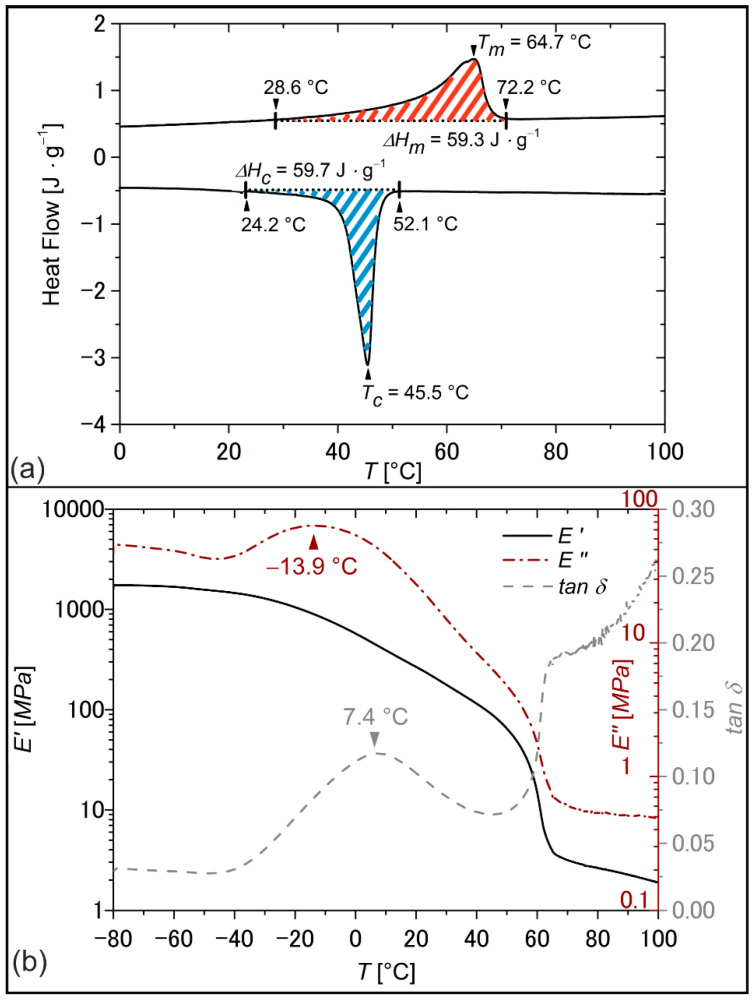
Thermal and thermomechanical properties of PDA-based PEU as determined by DSC (**a**), second heating and cooling with temperature rates of 10 °C·min^−1^, the enthalpies of melting *ΔH_m_*, red dashed area, and crystallization *ΔH_c_*, blue dashed area, are included) and DMA (**b**), the temperature dependence of storage modulus *E*′, loss modulus *E*″ and loss factor *tan δ* at a heating rate of 3 °C·min^−1^).

Having the good mechanical properties of the PEU in mind, differential scanning calorimetry (DSC) and dynamic mechanical analysis (DMA) were used to study the thermal phase transition behavior (Figure 5). This ensured that later thermomechanical tests were performed at appropriate temperatures. The calorimetric properties of the PEU were characterized by a broad melting transition between 29 °C and 72 °C and a crystallization transition spreading from 52 °C to 24 °C (Figure 5a), which were assigned to the phase transitions of PDA. In comparison with the measurement data provided for pure PDA (Figure 2), an extension of both phase transitions toward lower temperatures could be verified together with lower enthalpies of melting and crystallization. An inhibited crystallization behavior was expected because of the lower content of polyester polyol and phase segregation effects [47]. In the network structure of the PEU, the hard segments were efficiently acting as net points and were thus reducing the crystallinity of the soft segment as verified earlier for similar PEUs [48,49]. Apart from that, the elastic behavior as exemplified by the evolution of the storage modulus *E*′ exhibited a two-step decrease in the DMA (Figure 5b). This observation can be traced back to the consecutive devitrification and melting of the PDA phase. Indeed, such behavior is commonly observed for semicrystalline PEUs [50,51,52]. The presence of the hard segments acting as netpoints ensured that the PEU was still characterized by dimensional stability at temperatures exceeding the melting transition of the PDA phase at approximately 65 °C. In turn, the loss modulus *E*″, which is a measure for the viscous response of a polymer, exhibited a broad signal with a maximum at about −14 °C, which declined upon heating until a plateau formed at temperatures above 65 °C. The *tan δ* peak was located at 7 °C (Figure 5b). It is defined as the ratio between loss modulus *E*″ and storage modulus *E*′ and is often used to determine the glass transition temperature *T_g_* in urethane-based polymers [53,54].

**Figure 6:**
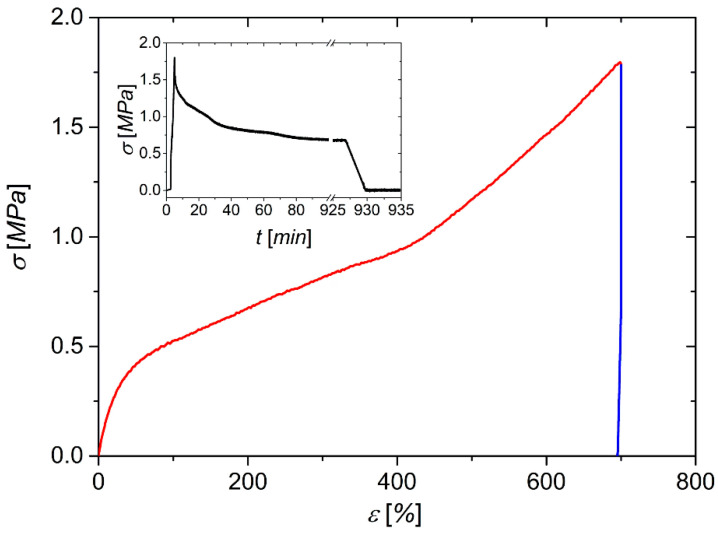
Stress–strain behavior of PDA-based PEU when programming the 2 W SME. The individual steps consisted of elongation at *T_d_* = 75 °C (red color, strain rate = 300%·min^−1^) followed by slow cooling and unloading at 23 °C (blue color, unloading rate = 1 N·min^−1^). The inset shows the evolution of stress over time for a longer period before unloading was carried out.

In the next step, a method for programming the 2W SME was developed in line with the thermal and mechanical properties of the PEU. Figure 6 shows the evolution of the stress–strain progression for the thermomechanical treatment of a type 5B tensile bar [43], which was obtained from FFF.

In accordance with the thermal behavior of the PEU, the tensile bar was heated to 75 °C and kept there for 20 min in order to ensure that all PDA crystals were molten before the polymer was elongated. In comparison to the stress–strain behavior verified in Figure 4, no yield point could be detected during elongation (Figure 6), and a significantly lower Young’s modulus of 1.4 ± 0.1 MPa was determined. A similar temperature dependence in stress–strain behavior is known from other physically cross-linked polymers like poly(1,4-butylene adipate)-based PEU [46]. However, to assure that the polymer chains were aligned in a highly oriented state, the strain was kept constant at a value of 700%, and the sample was slowly cooled to 23 °C in order to enable the extensive crystallization of the PDA phase before unloading was carried out. Although not verified in another experiment, the programming of the 2W SME appeared to be successful even at a much shorter crystallization time. This finding can be deduced from the inset of Figure 6, which shows that most of the stress relaxation took place immediately after stretching and that the recorded stress did not change significantly after a holding time of 100 min. Upon unloading, the polymer stabilized at a strain of 695%, which was close to the maximum strain applied.

**Figure 7:**
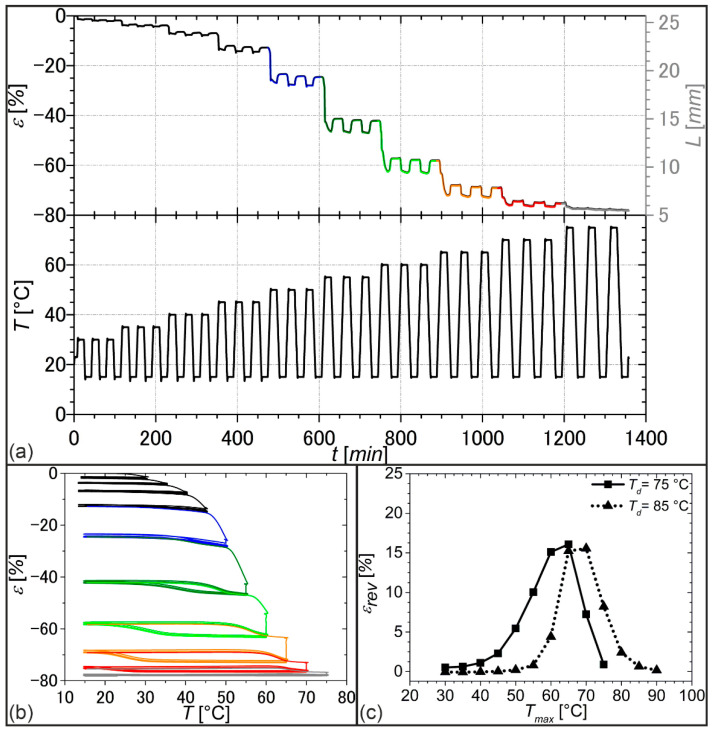
Influence of the selection of *T_max_* on actuation of PDA-based PEU under stress-free conditions: (**a**) evolution of strain *ε* and sample length *L* (differently colored according to the temperature intervals investigated) and temperature *T* with measuring time *t* for a sample deformed at *T_d_* = 75°; (**b**) strain–temperature relationship to the experiment shown in (**a**) using the same color codes; (**c**) evolution of thermoreversible strain *ε_rev_* depending on *T_max_* for two deformation temperatures, the values for *ε_rev_* are averaged for the second and third cycle.

Afterward, a DMA measurement was carried out on a sample, which was thermomechanically treated as reported before (Figure 6). The aim was to identify an ideal scenario for stress-free actuation by systematically varying the considered temperature range. The results are supplied in Figure 7.

Thermoreversible strain changes, here also denoted as actuation, could be detected in every single measurement cycle, even when continuously raising the upper temperature *T_max_* from 30 °C to 75 °C while keeping the lower temperature at 15 °C (Figure 7a,b). Apart from entropy elasticity, the phenomena of melting-induced contraction (MIC) and crystallization-induced elongation (CIE) were probably the main driving forces for actuation [19]. In fact, only a small quantity of crystallizable segments was present when selecting a lower maximum temperature *T_max_* because a larger part of the PDA phase was still in a crystalline state, resulting in weak elongation on cooling and weak contraction on heating, which can particularly be seen in the strain-temperature diagram of Figure 7b (black lines). By contrast, the successive increase in *T_max_* led first of all to an increase in the proportion of crystallizable segments because more PDA crystals were molten. In parallel, a hysteresis behavior was observed, and actuation substantially increased. The hysteresis was first observed when selecting a *T_max_* of 55 °C (Figure 7b, dark green color). Further increasing *T_max_* to 65 °C culminated in the most pronounced hysteresis (Figure 7b, orange color) with a maximum change in thermoreversible strain *ε*_*rev*_ of 16.1%. This becomes particularly clear in the associated *ε*_*rev*_*/T_max_* diagram (Figure 7c, solid line). Interestingly, the value for *T_max_* corresponded exactly with the melting peak temperature of the PDA phase in the DSC measurement (Figure 5a). The further increase of *T_max_* gave a decrease in actuation since the systematic melting of PDA crystals resulted in strain recovery of the PEU and thus a lower overall strain. In other words, the elongation at the beginning of each cooling step was gradually shifted to smaller values. Obviously, under these conditions, highly oriented crystals serving as netpoints were molten and could therefore no longer support the structural integrity associated with the respective morphological states of the polymer. 

Following a different approach, the programming route was modified by raising the deformation temperature *T_d_* to 85 °C, which was even further above the offset melting temperature of the crystalline PDA phase (Figure 5a). This time, a shift in the temperature range, in which actuation took place, could be witnessed (Figure 7c, dotted line). Indeed, the temperature region of actuation was raised together with the temperature, at which the maximum actuation occurred. Especially, in this case, we assume that PDA crystallites of higher temperature stability could be introduced in the course of deformation or directly afterward. These crystals obviously ensured the stability of the actuating polymer at elevated temperatures, namely in actuation states up to a temperature of about 80 °C. The rise in maximum actuation temperature at higher deformation temperature indicates that the temperature window of actuation can be adjusted by varying the deformation temperature and thus the programming conditions.

**Figure 8:**
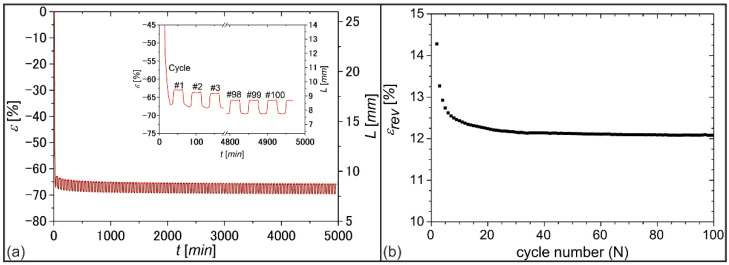
DMA measurement to determine the durability of actuation for PDA-based PEU: evolution of (**a**) nominal strain *ε* with time *t* and (**b**) thermoreversible strain *ε_rev_* with cycle number N.

To investigate the durability of actuation under stress-free conditions, a sample of PEU was programmed (*T_d_* = 75°) as described above and subjected to 100 heating-cooling cycles in the DMA with maximum and minimum temperatures of 64 °C and 15 °C, respectively (Figure 8).

In the beginning, the development of strain over time exhibited a strong drop in strain (Figure 8a), which was attributed to the melting of highly oriented PDA crystals. In particular, in the first five cycles, the actuation showed a drop (Figure 8b), which was presumably caused by rearrangements of polymer chains [55]. A more stable actuation was then observed; after about 25 cycles of heating and cooling, the actuation was nearly the same. Apparently, the PEU formed two temperature-bistable states, which differed in their elongation. In the end, *ε_rev_* approached an almost constant value of 12%.

**Figure 9:**
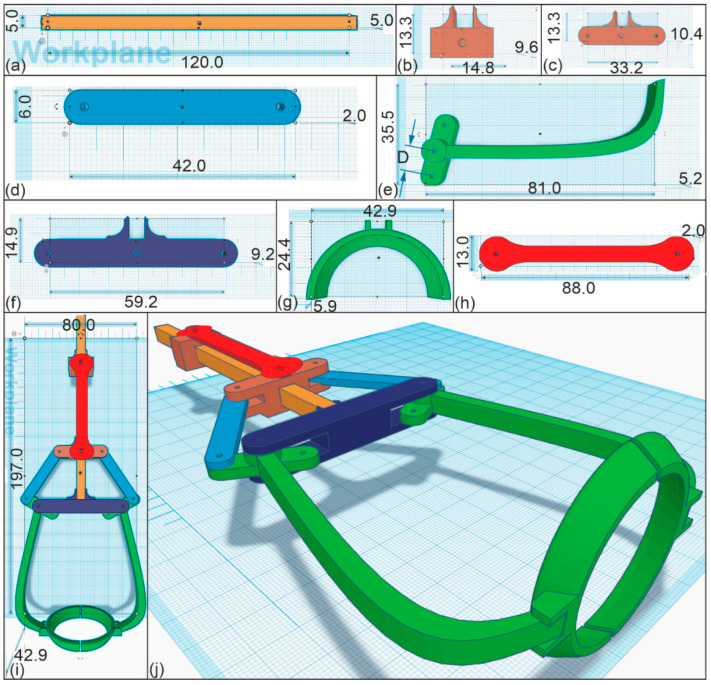
Technical drawing of a gripper: (**a**) main part; (**b**) upper actuator holder; (**c**) lower actuator-and linkage holder; (**d**) linkage bar; (**e**) gripper arm; (**f**) gripper holding part; (**g**) egg holder; (**h**) actuator element; (**i**) top view of assembled gripper arrangement; and (**j**) isometric view of gripper system. All data are provided in mm.

To take advantage of actuation, a suitable design for an actuator element made of PEU was developed, and possibilities for implementation into a gripping technology were explored using the linkage mechanism of Gholaminezhad et al. [56]. The corresponding computer-aided design (CAD) drawing of the gripper is shown in Figure 9. An important aspect of the design was that the base material of the gripper exhibited good mechanical stability, especially in the temperature range of actuation. Therefore polyethylene terephthalate glycol (PET-G) was selected as a rigid base material (Figure 9a–g) [57], while the centerpiece of the gripper, namely the actuator element, was made of thermomechanically pretreated PEU (*T_d_* = 75 °C, Figure 9h). The design of the gripper was chosen so that one millimeter of actuation was able to trigger a sixteen-fold increase in distance of the two gripper arms (Figure 9e). The diameter of the gripper element (Figure 9g) was set to 35 mm in order to enable that an object with the size of a hen’s egg could be reliably gripped. All parts exhibited in Figure 9 were manufactured via FFF. After rapid prototyping, the moving parts were assembled (Figure 9i,j), and care was taken to avoid friction between the individual components. 

**Figure 10:**
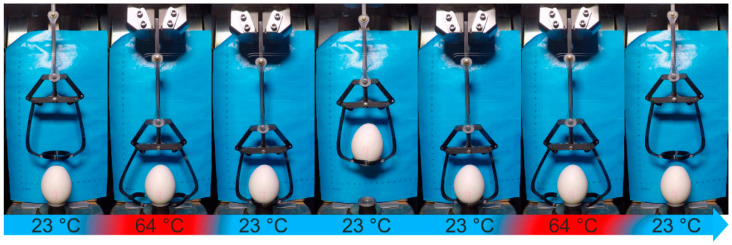
Gripper with PEU actuator element in operation, enabling the transport of a hen’s egg with a height of 55.5 mm, a width of 39 mm and a weight of 58 g. The lifting and lowering of the whole gripper were done manually.

The main part of the gripper (exhibited in Figure 9a) was constructed to hold the parts of the system either directly or indirectly. Once the actuator element with the PEU being in its low-temperature stable state was installed between the upper actuator holder and the lower actuator- and linkage holder (shown in Figure 9b,c), the actuation capability was studied (Figure 10). In this particular case, the minimum temperature was increased from 15 °C to 23 °C compared to the previous experiments in order to demonstrate a higher degree of practical suitability while the maximum temperature was kept constant at 64 °C.

As expected, the movement of the PEU could be transferred into motion perpendicular to the actuation direction in the form of a thermoreversible opening and closing of the clamps. Taking advantage of this behavior, a hen’s egg could be picked up, transported and deposited without causing any damage (Figure 10, s1.movie in Supplementary Materials).

In detail, raising the temperature to 64 °C resulted in the contraction of the PEU, which pulled the lower actuator- and linkage holder (exhibited in Figure 9c) and the linkage bar (exhibited in Figure 9d) upwards, thereby opening the gripper arms (shown in Figure 9e) with the attached egg holder (shown in Figure 9g). At this time, the gripper system was positioned near to the egg (second image in Figure 10). On cooling to 23 °C, the expansion of the PEU pushed the lower actuator and linkage holder (Figure 9c) and the linkage bar (Figure 9d) downwards, resulting in a closing of the gripper arms and the gripping of the egg. At this moment (middle of Figure 10), the object could be lifted at ambient temperature and taken to the desired location for further handling. On heating back to 64 °C, the gripper arms opened again, thus enabling the safe unloading of the fragile object (right side of Figure 10). Basically, the gripper system should be capable of repeated gripping and opening in a large number of cycles as indicated by the durability measurement (Figure 8).

**Figure 11:**
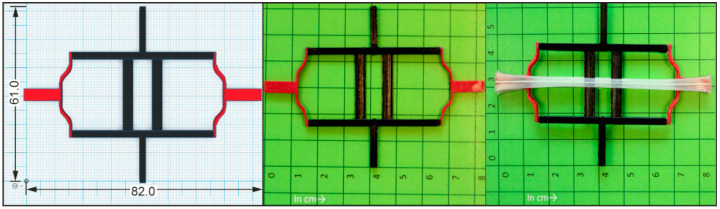
Unit cell consisting of an elastomeric material (Desmopan^®^ 9370AU, red color) and a stiff material (PET-G, black color): Technical drawing (**left**, all data are provided in mm.), additively manufactured unit cell (in the **middle**) and state after installing the actuator element (whitish color, **right**). Centimeter paper was used as a base in the second and third image.

In a fundamental approach, PEU actuator elements were integrated into distinct unit cells to obtain thermoresponsive programmable materials. Regarding the first concept, an actuator element made of PEU was fixed with glue on the surface of a unit cell to investigate to what extent its motion can be transferred to the cell. The unit cell was designed to allow larger deformation amplitudes between the temperature-dependent states of the PEU (Figure 11).

The cell was built up by the same PET-G as used for most parts of the gripper (black color) and by an elastic base material, here another thermoplastic polyurethane (red color), to which the PEU was attached with adhesive. The cell consisted of two center beams (black color), providing stability for outside buckling beams (red color). The cross beams were connecting all elements. This configuration of buckling beams can be used to provide metastable states, either being popped out or switched through. In this example, the beams were designed. Hence, in combination with the PEU actuator, the switched through the state could be reached at elevated temperature. Basically, such unit cells can be employed to reversibly absorb energy [58] or to implement mechanical memory behavior in materials [3].

**Figure 12:**
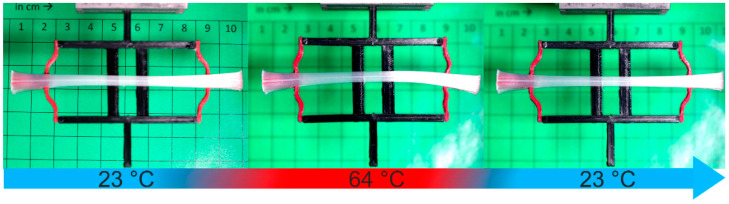
Thermoresponsiveness of a unit cell with a PEU actuator element. Centimeter paper was used as a base.

Upon heating and cooling between 23 °C and 64 °C, the actuator element was able to transfer its motion to the unit cell, resulting in a change of its structure with regard to the elastomer parts (Figure 12).

The PEU contracted on heating to 64 °C. As a result, the flexible parts of the unit cell were pulled together, which resulted in the formation of a second state whose stability was defined by the length of the actuator element (Figure 12, middle image). Cooling back to 23 °C was accompanied by the expansion of the PEU, leading to the return of the cell into its initial state (Figure 12, right image). This stretch-dominated approach makes clear that the movement of the actuator element could easily be transferred to the elastic parts of a cell, while the stiff parts of the cell remained unaffected. Thus, it could be demonstrated that combining a PEU actuator element with a unit cell allows switching back and forth between mechanical states, which differ in the shape of the elastic material.

**Figure 13:**
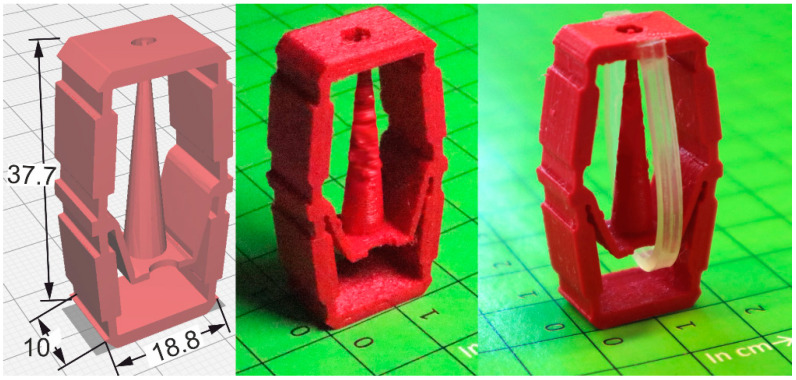
Unit cell with switchable surface topography. Technical drawing (**left**, all data are provided in mm.), unit cell after additive manufacturing with Desmopan^®^ 9370AU (**middle**) and unit cell as assembled with the PEU actuator element (**right**). Centimeter paper was used as a base in the second and third image.

As the last example, a macroscopic prototype of a unit cell for programmable wetting was produced. Normally such cells are roughly 100 µm high to work properly [59]. Nevertheless, a macroscopic prototype can be easily tested and scaled-down in a possible next step. The selected unit cell consisted of an outer cage providing potential contact to neighboring cells and mechanical stability for the inner mechanism. The outer cage contained a hole through which the spike can be pushed, leading to a strongly reduced contact area between the unit cell and, e.g., a drop of liquid on top (not shown here). The inner mechanisms allowed again two metastable states, similar to the previous case, with the spike being in or out. Therefore, a three-dimensional unit cell was manufactured from the same elastic base material as used above to autonomously open or close a hole in its surface depending on temperature (Figure 13). To achieve this, thermomechanically treated PEU was bent manually and attached to the unit cell with glue (Figure 13, right).

**Figure 14:**
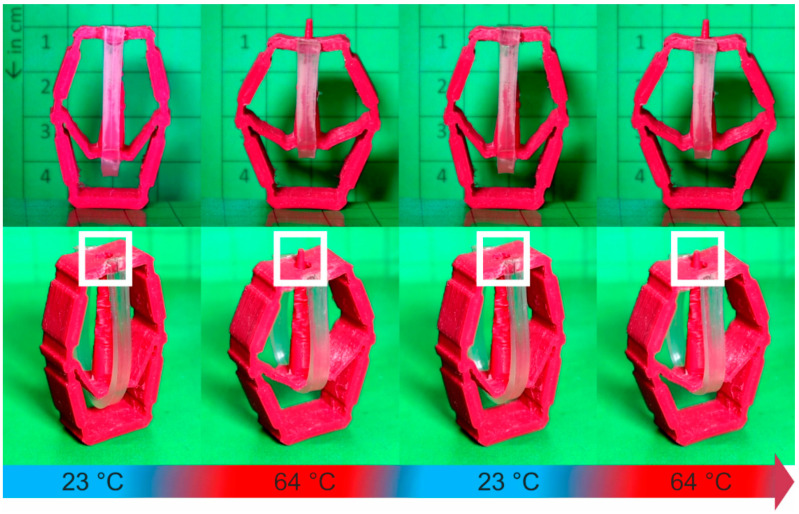
Thermoresponsiveness of a unit cell with a PEU actuator element. The system was cycled twice between 23 °C and 64 °C (**upper** row: front view, centimeter paper was used as background paper; **lower** row: isometric view, the white box was drawn in to illustrate the shape change in the surface).

Figure 14 shows the actuation of the unit cell upon twofold heating and cooling. First, the increase in temperature from 23 °C to 64 °C led to the contraction of the PEU, thus inducing an external strain on the unit cell and lifting the spike through the opening at the top plane. In parallel, the unit cell itself also changed its structure by broadening. In turn, cooling to 23 °C resulted in the expansion of the PEU actuator element, which led to the relaxation of the unit cell and thereby the retraction of the spike and the thinning of the cell. Following this bend-dominated approach, a proof of principle for the ability of the PEU to alter the structure of a complex unit cell and thereby the surface of its upper site could be achieved. A time-lapse video of unit cell actuation is provided as Supplementary Materials (s2.movie). The precise opening and closing of the hole in the surface was verified for in total four thermal cycles. However, due to the good durability of actuation (Figure 8), we assume that also, in this case, considerably more cycles can be run through.

## 3. Materials and Methods

### 3.1. Materials

1,10-Decanediol, 4,4′-methylene diphenyl diisocyanate (4,4′-MDI) and titanium(IV) isopropoxide (TTIP) were purchased from Fisher Scientific (Schwerte, Germany). For titration tests, acetic anhydride, methanol and potassium hydroxide solution in methanol with concentrations of 0.5 mol^−1^ and 0.1 mol^−1^ were purchased from Merck (Darmstadt, Germany). *N*-Methyl-2-pyrrolidone (2-NMP), chloroform and 4-dimethylaminopyridine (4-DMAP) were bought from Carl Roth (Karlsruhe, Germany). Adipic acid, 1,4-butanediol and a molecular sieve (4 Å) were obtained from Alfa Aesar (Kandel, Germany). A filament from polyethylene terephthalate glycol (PET-G) was purchased from FilamentWorld (Neu-Ulm, Germany). The ether-based thermoplastic polyurethane elastomer Desmopan^®^ 9370AU was supplied by Covestro Deutschland AG (Leverkusen, Germany). The polymer is characterized by a Shore A hardness of 70 [60] and was used as a flexible component in our unit cell approaches.

### 3.2. Synthesis of Polyester Diol

1,10-Decanediol and adipic acid were mixed at a molar ratio of 1.1:1 and heated in a three-necked round-bottomed flask, which was equipped with a mechanical stirrer, nitrogen gas inlet and distillation condenser. All reactants were molten at about 150 °C while titanium(IV) isopropoxide was added under stirring. Adjacently, the mixture was heated to 190 °C. After a remarkable decrease in distillation temperature, the mixture was further heated to 210 °C, whereupon the pressure was reduced to approximately 20 mbar. After two hours of continuous stirring, the melt was poured into a can. The obtained poly(1,10-decylene adipate) diol (PDA) solidified and was analyzed before PEU synthesis was carried out.

### 3.3. Titration

Titration was used to determine both the acid value and hydroxyl value and thus the number average molecular weight *M*_n_ of PDA. Therefore, a TitroLine 7000 from SI Analytics (Mainz, Germany) was employed. The procedure was executed in compliance with DIN EN ISO 2114 and 4629-2 [61,62]. To determine the acid value, a sample of PDA was dissolved in a mixture of chloroform/methanol with a volume ratio of 5:1. The solution was titrated against a potassium hydroxide solution in methanol, having a concentration of 0.1 mol^−1^. For the determination of the hydroxyl value, another sample of PDA was dissolved in chloroform. After adding acetic anhydride diluted in 2-NMP as well as 4-DMAP diluted in 2-NMP, the solution was heated and kept under stirring at 60 °C for 15 min. Thereafter, deionized water was added. After 12 min, the sample solution was titrated against a potassium hydroxide solution in methanol, having a concentration of 0.5 mol^−1^.

### 3.4. Synthesis of Polyester Urethane

A polyester urethane (PEU) was synthesized using the prepolymer method. In order to obtain a PEU with approximately 15% of hard segment content, the molar ratio of the reactants was set to 1:1.98:0.97with regard to PDA, 4,4′-MDI and 1,4-butanediol, respectively. The reaction was carried out with a slight excess of isocyanate (NCO/OH = 1.005). Overnight, PDA was dried in a glass reactor in a vacuum oven at 90 °C. The following day, it was heated under nitrogen flow and stirring to 120 °C. Adjacently, isocyanate was added, and the mixture was continuously stirred for 90 min. The obtained prepolymer was directly converted to PEU by adding 1,4-butanediol, serving as a chain extender. In parallel, the stirring speed was raised. As the viscosity increased significantly, the reaction was stopped, and the polymer melt was poured onto a plate covered with polytetrafluoroethylene. Finally, the PEU was cured in an oven for 120 min at 80 °C.

### 3.5. Fourier-Transform Infrared Spectroscopy

The synthesized PEU was investigated by Fourier-transform infrared (FT-IR) spectroscopy. The measurements were carried out with a Nicolet Nexus 470/670/870 FT-IR spectrometer from Thermo Fisher Scientific (Madison, WI, USA). The spectrometer was equipped with an attenuated total reflectance (ATR) device. In total, 40 scans with a spectral resolution of 2 cm^−1^ were averaged to give the spectrum from 4000 cm^−1^ to 650 cm^−1^.

### 3.6. Extrusion

**Figure 15:**
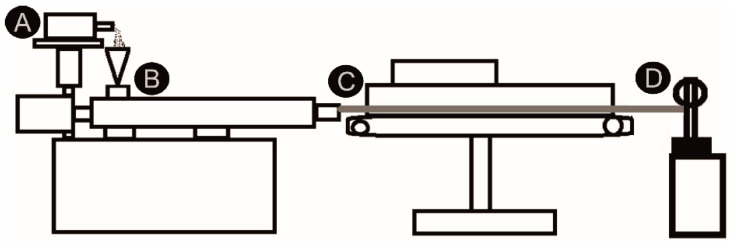
Technical drawing of an extrusion line as used for the production of PEU filaments: material feeding system (**A**), twin-screw extruder (**B**), conveyor belt (**C**) and filament winding machine (**D**). The extrudate is drawn in gray color.

The synthesized PEU was ground with a cutting mill type M 50/80 from Hellweg Maschinenbau (Roetgen, Germany). The obtained flakes were dried at 110 °C for 150 min in a vacuum drying chamber VDL 53 from Binder GmbH (Tuttlingen, Germany). Subsequently, the flakes were fed into an extrusion line to produce filaments. A schematic drawing of the extrusion line is shown in Figure 15.

The individual units of the extrusion line were put together in such a way that it included the volumetric material feeding system Color-exact 1000 from Plastic Recycling Machinery (Tranekær, Denmark), a Leistritz twin screw extruder MICRO 18 GL from Leistritz AG (Nürnberg, Germany), characterized by seven heating zones and a screw length of 600 mm, a conveyor belt, and a filament winder from Brabender GmbH and Co. KG (Duisburg, Germany). The temperatures of the individual heating zones of the extruder were 170 °C, 175 °C, 180 °C, 185 °C, 195 °C, 190 °C and 190 °C. To evaluate the quality of the filaments, the evolution in diameter was manually detected at regular intervals using a vernier caliper from Fowler High Precision (Newton, MA, USA).

### 3.7. Virtual Design and Fused Filament Fabrication

The online 3D modeling tinkercad.com [63], which is a web-based computer-aided design (CAD) program, was used for virtual construction. The developed CAD models were exported as standard triangle language (STL) files and later used for slicing.

After finalizing the designs of the tensile bar, actuator element, gripper and unit cells, Cura 3.6.1 [64] was used as a slicer program to generate numerically controlled codes, also denoted as G-codes. The 3D models were imported into the slicer program, and the models were sliced into layers according to the predefined printing parameters (Table 1). The specimens, as well as the actuator element for the gripper and for the different types of unit cells, were printed with the synthesized PDA-based PEU. Polyethylene terephthalate glycol (PET-G) was used to produce the rigid parts of the robotic gripper and one of the unit cells, while the thermoplastic polyurethane elastomer Desmopan^®^ 9370AU served as the elastic base material in both types of unit cells. The most relevant settings for additive manufacturing are listed in Table 1. To start additive manufacturing, the generated G-codes were transferred to the 3D printer. All 3D printed objects were produced by fused filament fabrication (FFF) using the commercially available 3D printer Ultimaker 3 from Ultimaker B.V. (Utrecht, The Netherlands).

### 3.8. Tensile Tests

The mechanical behavior of the synthesized PEU was investigated with the universal testing machine Criterion Model 43 from MTS (Eden Prairie, MN, USA). The device was equipped with a 500 N-load cell. The measurements were performed in compliance with DIN EN ISO 527 [43] by using dog-bone shaped tensile bars of type 5B as obtained from FFF. In the course of stretching, the velocity of 1%·min^−1^ was kept constant until a total strain of 5% was achieved before the specimen was further elongated with a velocity of 2000%·min^−1^ until rupture occurred. The Young’s modulus, which is defined as the slope of stress-strain evolution between two stress-strain points during elastic loading, was determined by linear regression for strain values below 2%. Every tensile test was repeated three times at ambient temperature.

### 3.9. Characterization of Thermal Properties

The phase transition behavior of PDA was characterized by differential scanning calorimetry (DSC) using a Q100 DSC from TA Instruments (New Castle, DE, USA). The measurements were conducted both with the synthesized PDA and PEU. In the case of the latter, the center part of an additively manufactured type 5B tensile bar [43] was examined. In general, samples with a weight of 5 mg were investigated.

The synthesized PDA was initially cooled to −30 °C before it was heated to 110 °C and cooled back to–30 °C. The thermal cycle was repeated to finalize the measurement. In contrast, a sample of PEU was thermally cycled between −50 °C and 225 °C. For cooling and heating, a rate of 10 °C·min^−^^1^ was applied. The temperature holding time at the minimum and maximum temperatures were 2 min.

### 3.10. Characterization of Thermomechanical Properties

The thermomechanical properties of the PEU were studied by dynamic mechanical analysis (DMA). The experiments were carried out with a Q800 DMA from TA Instruments (New Castle, DE, USA) using film tension clamps on multi-frequency–strain mode. A frequency of 10 Hz, a static force of 0.1 N and an oscillating amplitude of 10 µm were selected to investigate the center part of an additively manufactured type 5B tensile bar [43]. At first, the sample was cooled to −80 °C and held there for 5 min, before it was heated to 100 °C with a rate of 3 °C·min^−1^. In parallel, the evolution in storage modulus *E*′ and loss factor *tan δ* was determined.

### 3.11. Programming and Characterization of 2W-SME

The programming of PEU samples was conducted with an MTS Criterion universal testing machine (model 43) from MTS Systems Corporation (Eden Prairie, MN, USA). The device was equipped with a 500 N-load cell and was operated with a temperature chamber, which was controlled by a Eurotherm temperature controller unit. Two heating elements were located at the back of the chamber. Liquid nitrogen from a Dewar’s vessel was fed into the chamber under a pressure of 1.3 bar as an essential prerequisite for cooling. At the beginning of thermomechanical treatment, the thermochamber was preheated to the deformation temperature *T_d_*, which was either 75 °C or 85 °C, and the specimen was fixed in the pneumatic clamps of the universal testing machine using a clamping air pressure of 0.4 bar. After 20 min at *T_d_*, the specimen was elongated to a maximum tensile strain of 700% using a rate of 300%·min^−1^. In the next step, the heating elements of the temperature chamber were switched off, whereupon the specimen cooled down slowly to 23 °C while the clamping distance was kept constant. After unloading with a rate of 1 N·min^−1^, the sample was removed from the clamps.

The Q800 DMA from TA Instruments (New Castle, DE, USA) was utilized to investigate the two-way shape memory properties of the PEU. For this purpose, a cuboid centerpiece of a specimen, which was thermomechanically treated as described above (*T_d_* = 75 °C) and characterized by a dimensioning of 25 mm × 0.9 mm × 0.8 mm, was cut out and fixed in the film tension clamps of the DMA device. The actuation of the PEU was adjacently studied under stress-free conditions by cycling the temperature. Against this background, a first test series was run, in which the maximum temperature *T_max_* was systematically varied, and a constant minimum temperature *T_min_* of 15 °C was selected. For this purpose, a sample, which was thermomechanically treated as described above (*T_d_* = 75 °C), was heated from 23 °C to *T_max_* = 30 °C and held there for 15 min, before it was cooled to 15 °C, at which the temperature was kept for another 15 min. The heating and cooling were carried out three times. Afterward, the three cycles were repeated for each temperature *T_max_* between 30 °C and 75 °C with an increment of 5 °C. Heating and cooling rates of 5 °C·min^−1^ were used for all experiments conducted.

In another approach, a cutout of a specimen, which was deformed at 85 °C, was investigated under the same conditions, but with maximum temperatures *T_max_* between 30 °C and 90 °C.

In a durability experiment, a cutout of a thermomechanically treated PEU specimen (*T_d_* = 75 °C) was studied under stress-free conditions by cycling the temperature between 15 °C and 64 °C. This time, actuation was investigated in 100 thermal cycles.

The reversible strain *ε_rev_* is the key parameter when studying the actuation of polymers. It can be defined according to Equation (1):

(1) $$\varepsilon_{rev}\left(N\right)=\;\frac{l_{low}\left(N\right)-l_{high}\left(N\right)}{l_{high}\left(N\right)}\;\times \;100\%$$

Herein, *l_low_*(*N*) and *l_high_*(*N*) are the lengths of the specimen in the *N*th cycle of actuation at the respective temperatures *T_low_* and *T_high_*.

### 3.12. Demonstrator Development

Samples of PEU were additively manufactured by means of FFF, characterized by a size of 28 mm × 7 mm × 5 mm. Subsequently, they were thermomechanically treated with the procedure described in Section 3.11 (*T_d_* = 75 °C) to obtain the desired actuator elements. The actuation of the gripper was studied in the temperature chamber of our MTS Criterion universal testing machine (model 43) from MTS Systems Corporation (Eden Prairie, MN, USA). Solvent-free superglue from Pattex [65] was used to attach the actuator elements to the unit cells. For functional testing, the unit cells were characterized similarly as the gripper in the temperature chamber of our universal testing machine. To better visualize actuation, the unit cells were placed on a platform, which was lined with centimeter paper. Both in case of the gripper and the unit cells, the temperature was cycled in between 23 °C and 64 °C with heating and cooling rates of 5 °C·min^−1^. 

## 4. Conclusions

A novel polyester urethane (PEU) was synthesized. After processing and thermomechanical treatment, thermoreversible shape changes could be witnessed. The PEU was used as an actuator element in a gripper, which was designed to precisely convert the comparatively small change in the shape of a few millimeters into a macroscopically well visible and technically relevant motion. The cautious gripping, holding and releasing of a hen’s egg qualified the gripper for applications in soft robotics. Compared to grippers made entirely of shape memory polymer, the introduced concept has the advantage that the materials that come into contact with an object to be gripped can be freely selected according to the design of the gripper and that predefined movements can be carried out. Hence, in the end, a high degree of system control is possible. Future developments in gripper design are able to expand the range of possibilities, e.g., to grip even more challenging and bigger objects. The design space thus created allows the production of completely new systems with programmable gripping, holding and releasing properties.

Moreover, the implementation of the PEU actuator into macroscopic unit cells with elastic components led to programmable materials, which moved autonomously as a function of temperature. It is precisely this behavior that can initiate a paradigm shift in the future, in which the programming of material is understood as the programming of a functionality. The internal structure of materials is such that the material properties and behavior change reversibly according to a program. This is achieved by programming the reaction of the material to temperature signals into the material structure. In this way, completely new components with specific properties can be produced, which can be used in a wide variety of contexts. Considering that such concepts require neither control electronics nor cables or other technical devices, the self-sufficient material behavior is all the more promising.

## Figures and Tables

**Table 1:** Printing parameters selected for additive manufacturing with different materials.

	PET-G	PEU	Desmopan^®^ 9370AU
Diameter of the nozzle (µm)	400	400	400
Temperature of the nozzle (°C)	235	208	225
Speed of print head (mm·s^−1^)	50	15	25
Build platform temperature (°C) Build platform temperature (°C)	85	75	60
Layer height (mm)	0.1	0.1	0.1

## Data Availability

The data presented in this study are available in Supplementary Materials.

## Supplementary Materials

The following materials are available online, Videos: s1.movie: Actuation of the PEU-based gripper system (Figure 10) and s2.movie: Actuation of PEU to achieve unit cell morphing (Figure 14). Figure S1: FT-IR spectrum of PDA-based PEU.

## Sample Availability

Samples of the compounds are provided by the authors in special cases.

## References

[1] Lee J.-H., Singer J.P., Thomas E.L. (2012). Micro-/nanostructured mechanical metamaterials. Adv. Mater. Weinheim..

[2] Yu X., Zhou J., Liang H., Jiang Z., Wu L. (2018). Mechanical metamaterials associated with stiffness, rigidity and compressibility: A brief review. Prog. Mater. Sci..

[3] Fischer S.C.L., Hillen L., Eberl C. (2020). Mechanical Metamaterials on the Way from Laboratory Scale to Industrial Applications: Challenges for Characterization and Scalability. Materials.

[4] Liu C., Qin H., Mather P.T. (2007). Review of progress in shape-memory polymers. J. Mater. Chem..

[5] Holme D. (1806). A Description of a Property of Caoutchouc, or Indian Rubber; with some Reflections on the Cause of the Elasticity of this Substance. Philos. Mag..

[6] Huang W.M., Ding Z., Wang C.C., Wei J., Zhao Y., Purnawali H. (2010). Shape memory materials. Mater. Today.

[7] Small W., Singhal P., Wilson T.S., Maitland D.J. (2010). Biomedical applications of thermally activated shape memory polymers. J. Mater. Chem..

[8] Pretsch T. (2010). Review on the Functional Determinants and Durability of Shape Memory Polymers. Polymers.

[9] Peponi L., Navarro-Baena I., Kenny J.M., Aguilar M.R., San Román J. (2014). 7—Shape memory polymers: Properties, synthesis and applications. Smart Polymers and Their Applications.

[10] Pilate F., Toncheva A., Dubois P., Raquez J.-M. (2016). Shape-memory polymers for multiple applications in the materials world. Eur. Polym. J..

[11] Karger-Kocsis J., Kéki S. (2017). Review of Progress in Shape Memory Epoxies and Their Composites. Polymers.

[12] Sun L., Wang T.X., Chen H.M., Salvekar A.V., Naveen B.S., Xu Q., Weng Y., Guo X., Chen Y., Huang W.M. (2019). A Brief Review of the Shape Memory Phenomena in Polymers and Their Typical Sensor Applications. Polymers.

[13] Cho J.W., Kim J.W., Jung Y.C., Goo N.S. (2005). Electroactive Shape-Memory Polyurethane Composites Incorporating Carbon Nanotubes. Macromol. Rapid Commun..

[14] Leng J., Lan X., Liu Y., Du S. (2009). Electroactive thermoset shape memory polymer nanocomposite filled with nanocarbon powders. Smart Mater. Struct..

[15] Mohr R., Kratz K., Weigel T., Lucka-Gabor M., Moneke M., Lendlein A. (2006). Initiation of shape-memory effect by inductive heating of magnetic nanoparticles in thermoplastic polymers. Proc. Natl. Acad. Sci. USA.

[16] Schmidt A.M. (2006). Electromagnetic Activation of Shape Memory Polymer Networks Containing Magnetic Nanoparticles. Macromol. Rapid Commun..

[17] Fang T., Cao L., Chen S., Fang J., Zhou J., Fang L., Lu C., Xu Z. (2018). Preparation and assembly of five photoresponsive polymers to achieve complex light-induced shape deformations. Mater. Des..

[18] Wang H., Fang L., Zhang Z., Epaarachchi J., Li L., Hu X., Lu C., Xu Z. (2019). Light-induced rare earth organic complex/shape-memory polymer composites with high strength and luminescence based on hydrogen bonding. Compos. Part A Appl. Sci. Manuf..

[19] Chung T., Romo-Uribe A., Mather P.T. (2008). Two-Way Reversible Shape Memory in a Semicrystalline Network. Macromolecules.

[20] Behl M., Kratz K., Zotzmann J., Nöchel U., Lendlein A. (2013). Reversible bidirectional shape-memory polymers. Adv. Mater. Weinheim..

[21] Bothe M., Pretsch T. (2013). Bidirectional actuation of a thermoplastic polyurethane elastomer. J. Mater. Chem. A.

[22] Meng Y., Jiang J., Anthamatten M. (2015). Shape Actuation via Internal Stress-Induced Crystallization of Dual-Cure Networks. ACS Macro Lett..

[23] Meng Y., Yang J.-C., Lewis C.L., Jiang J., Anthamatten M. (2016). Photoinscription of Chain Anisotropy into Polymer Networks. Macromolecules.

[24] Bothe M., Pretsch T. (2012). Two-Way Shape Changes of a Shape-Memory Poly(ester urethane). Macromol. Chem. Phys..

[25] Walter M., Friess F., Krus M., Zolanvari S.M.H., Grün G., Kröber H., Pretsch T. (2020). Shape Memory Polymer Foam with Programmable Apertures. Polymers.

[26] Rus D., Tolley M.T. (2015). Design, fabrication and control of soft robots. Nature.

[27] Zolfagharian A., Kouzani A.Z., Khoo S.Y., Moghadam A.A.A., Gibson I., Kaynak A. (2016). Evolution of 3D printed soft actuators. Sens. Actuators A Phys..

[28] Shintake J., Cacucciolo V., Floreano D., Shea H. (2018). Soft Robotic Grippers. Adv. Mater. Weinheim..

[29] Chen T., Bilal O.R., Shea K., Daraio C. (2018). Harnessing bistability for directional propulsion of soft, untethered robots. Proc. Natl. Acad. Sci. USA.

[30] Ma H., Xiao X., Zhang X., Liu K. (2020). Recent advances for phase-transition materials for actuators. J. Appl. Phys..

[31] Scalet G. (2020). Two-Way and Multiple-Way Shape Memory Polymers for Soft Robotics: An Overview. Actuators.

[32] Chen Y., Chen C., Rehman H.U., Zheng X., Li H., Liu H., Hedenqvist M.S. (2020). Shape-Memory Polymeric Artificial Muscles: Mechanisms, Applications and Challenges. Molecules.

[33] Tolley M.T., Felton S.M., Miyashita S., Xu L., Shin B., Zhou M., Rus D., Wood R.J. Self-folding shape memory laminates for automated fabrication. Proceedings of the 2013 IEEE/RSJ International Conference on Intelligent Robots and Systems.

[34] Ge Q., Sakhaei A.H., Lee H., Dunn C.K., Fang N.X., Dunn M.L. (2016). Multimaterial 4D Printing with Tailorable Shape Memory Polymers. Sci. Rep..

[35] Yang Y., Chen Y., Li Y., Chen M.Z.Q., Wei Y. (2017). Bioinspired Robotic Fingers Based on Pneumatic Actuator and 3D Printing of Smart Material. Soft Robot..

[36] Zhou J., Turner S.A., Brosnan S.M., Li Q., Carrillo J.-M.Y., Nykypanchuk D., Gang O., Ashby V.S., Dobrynin A.V., Sheiko S.S. (2014). Shapeshifting: Reversible Shape Memory in Semicrystalline Elastomers. Macromolecules.

[37] Kadic M., Milton G.W., van Hecke M., Wegener M. (2019). 3D metamaterials. Nat. Rev. Phys..

[38] Vouyiouka S.N., Topakas E., Katsini A., Papaspyrides C.D., Christakopoulos P. (2013). A Green Route for the Preparation of Aliphatic Polyesters via Lipase-catalyzed Prepolymerization and Low-temperature Postpolymerization. Macromol. Mater. Eng..

[39] Kim S.G., Lee D.S. (2002). Effect of polymerization procedure on thermal and mechanical properties of polyether based thermoplastic polyurethanes. Macromol. Res..

[40] Yilgör I., Yilgör E., Wilkes G.L. (2015). Critical parameters in designing segmented polyurethanes and their effect on morphology and properties: A comprehensive review. Polymer.

[41] Kasprzyk P., Sadowska E., Datta J. (2019). Investigation of Thermoplastic Polyurethanes Synthesized via Two Different Prepolymers. J. Polym. Environ..

[42] Chalissery D., Pretsch T., Staub S., Andrä H. (2019). Additive Manufacturing of Information Carriers Based on Shape Memory Polyester Urethane. Polymers.

[43] DIN EN ISO 527-2-1996-07-Beuth.de. https://www.beuth.de/en/standard/din-en-iso-527-2/2808009.

[44] Bothe M., Mya K.Y., Jie Lin E.M., Yeo C.C., Lu X., He C., Pretsch T. (2012). Triple-shape properties of star-shaped POSS-polycaprolactone polyurethane networks. Soft Matter.

[45] Huang W.M., Lu H.B., Zhao Y., Ding Z., Wang C.C., Zhang J.L., Sun L., Fu J., Gao X.Y. (2014). Instability/collapse of polymeric materials and their structures in stimulus-induced shape/surface morphology switching. Mater. Des..

[46] Mirtschin N., Pretsch T. (2015). Designing temperature-memory effects in semicrystalline polyurethane. RSC Adv..

[47] van Horn R.M., Steffen M.R., O’Connor D. (2018). Recent progress in block copolymer crystallization. Polym. Cryst..

[48] Li F., Hou J., Zhu W., Zhang X., Xu M., Luo X., Ma D., Kim B.K. (1996). Crystallinity and morphology of segmented polyurethanes with different soft-segment length. J. Appl. Polym. Sci..

[49] Bogdanov B., Toncheva V., Schacht E., Finelli L., Sarti B., Scandola M. (1999). Physical properties of poly(ester-urethanes) prepared from different molar mass polycaprolactone-diols. Polymer.

[50] Chen S., Hu J., Liu Y., Liem H., Zhu Y., Meng Q. (2007). Effect of molecular weight on shape memory behavior in polyurethane films. Polym. Int..

[51] Chen S., Hu J., Liu Y., Liem H., Zhu Y., Liu Y. (2007). Effect of SSL and HSC on morphology and properties of PHA based SMPU synthesized by bulk polymerization method. J. Polym. Sci. Part B Polym. Phys..

[52] Bothe M., Emmerling F., Pretsch T. (2013). Poly(ester urethane) with Varying Polyester Chain Length: Polymorphism and Shape-Memory Behavior. Macromol. Chem. Phys..

[53] Rocco J.A.F.F., Lima J.E.S., Lourenço V.L., Batista N.L., Botelho E.C., Iha K. (2012). Dynamic mechanical properties for polyurethane elastomers applied in elastomeric mortar. J. Appl. Polym. Sci..

[54] Li J., Kan Q., Chen K., Liang Z., Kang G. (2019). In Situ Observation on Rate-Dependent Strain Localization of Thermo-Induced Shape Memory Polyurethane. Polymers.

[55] Lu L., Cao J., Li G. (2018). Giant reversible elongation upon cooling and contraction upon heating for a crosslinked cis poly(1,4-butadiene) system at temperatures below zero Celsius. Sci. Rep..

[56] Gholaminezhad I., Jamali A., Assimi H. (2017). Multi-objective reliability-based robust design optimization of robot gripper mechanism with probabilistically uncertain parameters. Neural. Comput. Appl..

[57] Latko-Durałek P., Dydek K., Boczkowska A. (2019). Thermal, Rheological and Mechanical Properties of PETG/rPETG Blends. J. Polym. Environ..

[58] Shan S., Kang S.H., Raney J.R., Wang P., Fang L., Candido F., Lewis J.A., Bertoldi K. (2015). Multistable Architected Materials for Trapping Elastic Strain Energy. Adv. Mater. Weinheim..

[59] Specht M., Berwind M., Eberl C. (2020). Adaptive Wettability of a Programmable Metasurface. Adv. Eng. Mater..

[60] M-Base Engineering + Software GmbH. CAMPUSplastics|Datenblatt Desmopan 9370A. https://www.campusplastics.com/campus/de/datasheet/Desmopan%C2%AE+9370A/Covestro+Deutschland+AG/22/15fed464.

[61] Deutsches Institut für Normung e.V (2006). Kunststoffe (Polyester) und Beschichtungsstoffe (Bindemittel)—Bestimmung der partiellen Säurezahl und der Gesamtsäurezahl.

[62] Deutsches Institut für Normung e.V (2016). Bindemittel für Beschichtungsstoffe—Bestimmung der Hydroxylzahl—Teil 2: Titrimetrisches Verfahren mit Katalysator.

[63] Dashboard|Tinkercad. https://www.tinkercad.com/dashboard.

[64] Ultimaker Cura: Powerful, Easy-To-Use 3D Printing Software. https://ultimaker.com/software/ultimaker-cura.

[65] Flüssig Mini Trio. https://www.pattex.de/de/products/klebstoff/sekundenkleber/fluessig_mini_trio.html.

