# Supplementary material for: Actuating Shape Memory Polymer for Thermoresponsive Soft Robotic Gripper and Programmable Materials
Source: Molecules. 2021 Jan 20;26(3):522. doi: 10.3390/molecules26030522 (PMC7864034; doi:10.3390/molecules26030522)
Supplement: Supplementary file 1 [file molecules-26-00522-s001.zip › molecules-1043548-supplementary-final/molecules-1043548-supplementary-final.pdf]

Supplementary Material

Fourier-transform infrared (FT-IR) spectroscopy

Normally, a distinct signal at about 2270 cm^{-1} occurs in the FT-IR spectrum of polyurethanes in case of an incomplete reaction as associated with freely available isocyanate groups [1,2]. In the present case, the reaction seemed to be almost complete because the signal at 2283 cm^{-1} was very weak, while at the same time, vibration modes were present as characteristic for the formation of a polyester urethane (Figure S1) [3].

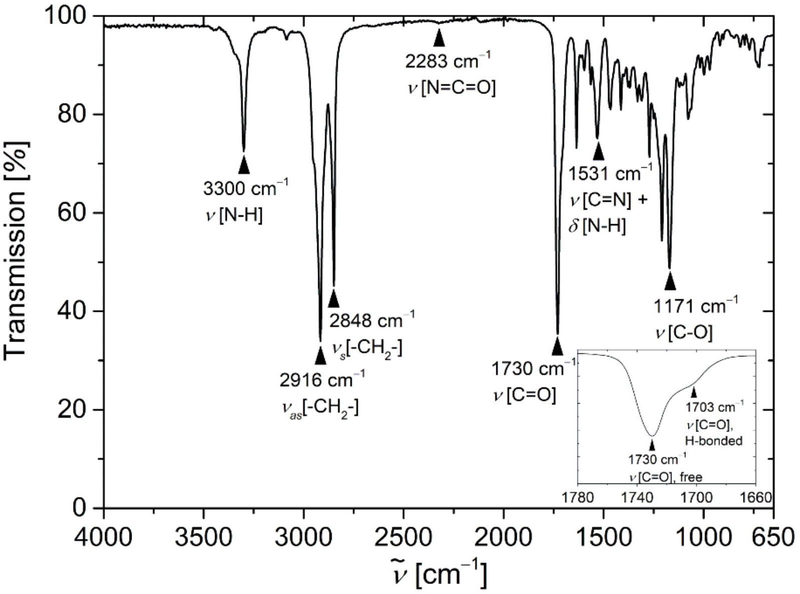

Figure S1. FT-IR spectrum of poly(1,10-decylene adipate) diol (PDA)-based polyester urethane (PEU), including the assignment of vibration modes and the specification of their wavenumbers. The inset highlights the carbonyl stretching vibration region.

In fact, a more detailed analysis of the FT-IR spectrum showed distinct absorbances at 2916 cm^{-1} and 2848 cm^{-1}, which can be assigned to the asymmetric and symmetric stretching vibrations of CH_2 entities [2,4,5]. Two overlapping bands are present in the carbonyl stretching region – one dominating peak apparently centered at 1730 cm^{-1} and a broad shoulder at 1703 cm^{-1}. The two signals are ascribed to the stretching vibrations of free (non-hydrogen-bonded) and hydrogen-bonded carbonyl, respectively [3,6–8]. The band at 1171 cm^{-1} was assigned to the corresponding ν[C–O] stretching vibrations [4,9,10]. The stretching vibration ν[N–H] at 3300 cm^{-1} and an amide peak (ν[C=N] + δ[N–H]) at 1531 cm^{-1} were attributed to vibrations associated with the hard segments of the PEU [11,12].

References

1. Sáenz-Pérez, M.; Lizundia, E.; Laza, J.M.; García-Barrasa, J.; Vilas, J.L.; León, L.M. Methylene diphenyl diisocyanate (MDI) and toluene diisocyanate (TDI) based polyurethanes: thermal, shape-memory and mechanical behavior. *Rsc Adv.* 2016, *6*, 69094–69102, doi:10.1039/c6ra13492k.
2. Tan, C.; Tirri, T.; Wilen, C.-E. Investigation on the Influence of Chain Extenders on the Performance of One-Component Moisture-Curable Polyurethane Adhesives. *Polym. (Basel)* 2017, *9*, doi:10.3390/polym9050184.
3. Pretsch, T.; Jakob, I.; Müller, W. Hydrolytic degradation and functional stability of a segmented shape memory poly(ester urethane). *Polym. Degrad. Stab.* 2009, *94*, 61–73, doi:10.1016/j.polymdegradstab.2008.10.012.

4. Panwiriyarat, W.; Tanrattanakul, V.; Pilard, J.-F.; Pasetto, P.; Khaokong, C. Effect of the diisocyanate structure and the molecular weight of diols on bio-based polyurethanes. *J. Appl. Polym. Sci.* 2013, *130*, 453–462, doi:10.1002/app.39170.
5. Lei, W.; Fang, C.; Zhou, X.; Cheng, Y.; Yang, R.; Liu, D. Morphology and thermal properties of polyurethane elastomer based on representative structural chain extenders. *Thermochim. Acta* 2017, *653*, 116–125, doi:10.1016/j.tca.2017.04.008.
6. Seymour, R.W.; Estes, G.M.; Cooper, S.L. Infrared Studies of Segmented Polyurethan Elastomers. I. Hydrogen Bonding. *Macromolecules* 1970, *3*, 579–583, doi:10.1021/ma60017a021.
7. Lee, H.S.; Wang, Y.K.; Hsu, S.L. Spectroscopic analysis of phase separation behavior of model polyurethanes. *Macromolecules* 1987, *20*, 2089–2095, doi:10.1021/ma00175a008.
8. Teo, L.-S.; Chen, C.-Y.; Kuo, J.-F. Fourier Transform Infrared Spectroscopy Study on Effects of Temperature on Hydrogen Bonding in Amine-Containing Polyurethanes and Poly(urethane−urea)s. *Macromolecules* 1997, *30*, 1793–1799, doi:10.1021/ma961035f.
9. Yen, M.-S.; Cheng, K.-L. Synthesis and physical properties of H 12 MDI-based polyurethane resins. *J Polym. Res.* 1996, *3*, 115–123, doi:10.1007/BF01492902.
10. Peruzzo, P.J.; Anbinder, P.S.; Pardini, O.R.; Vega, J.R.; Amalvy, J.I. Influence of diisocyanate structure on the morphology and properties of waterborne polyurethane-acrylates. *Polym. J.* 2012, *44*, 232–239, doi:10.1038/pj.2011.111.
11. Schoonover, J.R.; Thompson, D.G.; Osborn, J.C.; ORLER, E.B.; Wrobleski, D.A. Infrared linear dichroism study of a hydrolytically degraded poly(ester urethane). In *Polymer degradation and stability*, 2001; pp. 87–96, ISBN 0141-3910.
12. Schoonover, J.R.; Steckle, W.P.; Cox, J.D.; Johnston, C.T.; Wang, Y.; Gillikin, A.M.; Palmer, R.A. Humidity-dependent dynamic infrared linear dichroism study of a poly(ester urethane). *Spectrochim. Acta Part A: Mol. Biomol. Spectrosc.* 2007, *67*, 208–213, doi:10.1016/j.saa.2006.07.015.
